# Experimental Research on Heat Transfer Through 3D-Printed Plates: Implications for the Development of Smart Facades

**DOI:** 10.3390/ma19132793

**Published:** 2026-07-01

**Authors:** Dan-Radu Baraboi, Daniela Șova, Gabriel Năstase

**Affiliations:** 1 Department of Building Services, Transylvania University of Brasov, 500036 Brasov, Romania; gabrielnastase@unitbv.ro; 2 Department of Mechanical Engineering, Transylvania University of Brasov, 500036 Brasov, Romania; sova.d@unitbv.ro

**Keywords:** 3D printing, fused deposition modeling (FDM), effective thermal conductivity, apparent thermal conductivity, guarded hot plate (GHP), smart facades, PET-CF, PLA Aero, thermal resistance, polymer composites

## Abstract

To address the increasing demand for energy-efficient buildings, this study experimentally characterizes the effective (λ_eff_) and apparent (λ_app_) thermal conductivity of 3D-printed polymer plates. While 3D printing offers significant design flexibility, a lack of comprehensive comparative data between printable polymers and conventional building materials limits their integration into large-scale facade systems. This research investigates four distinct materials: standard polylactic acid (PLA Basic), foamable poly-L-lactic acid (PLA Aero), amorphous polyethylene terephthalate glycol (PETG), and carbon fiber-reinforced polyethylene terephthalate (PET-CF). Utilizing the guarded hot plate (GHP) method (ASTM C177, EN 12667, EN 12939), steady-state heat flux and temperature gradients were measured. The methodology incorporates a rigorous uncertainty analysis (k = 2) addressing the inherent inhomogeneity of additively manufactured components. Results demonstrate significant variations: PLA Aero achieved a 57.3% reduction in thermal conductivity (0.114 ± 0.005 W/(m·K)) compared to PLA Basic (0.267 ± 0.011 W/(m·K)), while PET-CF showed increased conductivity (0.533 ± 0.021 W/(m·K)) due to carbon fiber bridging. Notably, multi-layered PLA Aero assemblies outperformed conventional double-glazed units, reaching a minimum λ_app_ of 0.051 W/(m·K). These findings validate the GHP method for 3D-printed polymers and provide a technical foundation for material selection in next-generation, energy-efficient smart facades.

## 1. Introduction

Buildings account for approximately 40% of global energy consumption and 36% of CO2 emissions within the EU [1]. This significant environmental footprint drives the urgent demand for sustainable construction strategies [2] and reduced operational energy use [3], as mandated by Nearly Zero-Energy Building (NZEB) and Zero-Emission Building (ZEB) directives.

As the primary interface between the internal and external environments [4], the building facade fundamentally dictates energy demand. Consequently, transitioning from traditional static designs—characterized by fixed thermophysical and optical properties, such as masonry walls or single-layered insulation boards [5]—to dynamic smart facades [6] has become imperative. Unlike conventional envelopes, smart facades can adapt to fluctuating environmental conditions, offering a pathway toward achieving high-performance building standards.

**Figure 1:**
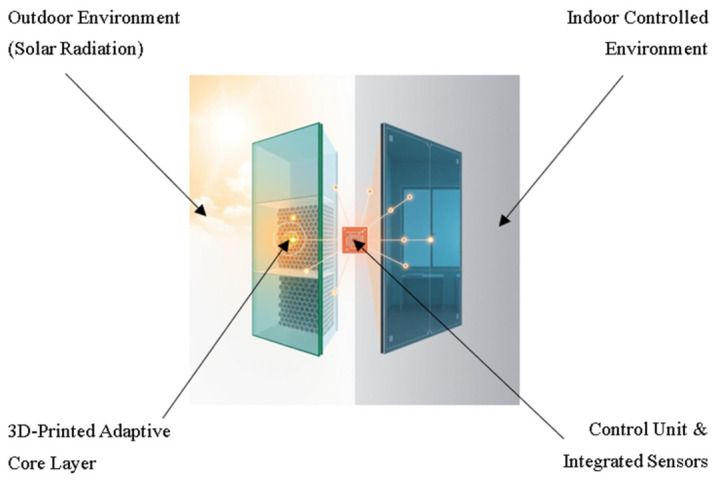
Conceptual framework of a smart facade system. The illustration shows the potential integration of a 3D-printed cellular core (for optimized thermal resistance) with a sensor network and control unit designed to manage heat transfer between the external solar-exposed environment and the internal conditioned space. Note: This diagram represents a contextual motivation for smart facade development; the current study focus is limited to the experimental characterization of the passive thermal properties of the printed components.

Smart facades integrate advanced technologies (sensors, controls) to adapt to environmental conditions [7], actively managing energy flows, ventilation, and indoor air quality. A conceptual diagram of a smart facade system is provided in Figure 1, illustrating the potential integration of 3D-printed components with active sensors. This approach aims to provide a foundation for reducing operational energy consumption [8] and enhances comfort, marking a major step towards sustainable buildings [9]. While Figure 1 presents an active smart facade concept, it should be noted that the present experimental work focuses exclusively on the passive thermal performance of 3D-printed specimens.

Developing smart facades critically relies on material innovation. 3D printing emerges as a disruptive technology, enabling complex geometries and optimized thermal properties [10]. However, for 3D-printed smart facades to reach full potential, understanding material thermophysical properties [11] is crucial. It is essential to distinguish between effective thermal conductivity, applicable to monolithic materials, and apparent thermal conductivity, which describes the performance of complex or multi-layered assemblies [12]. The present study addresses this by experimentally investigating at a laboratory scale the preliminary thermal performance of 3D-printed plates from polylactic acid (PLA Basic), poly-L-lactic acid (PLA Aero), polyethylene terephthalate glycol (PETG), and carbon fiber-reinforced polyethylene terephthalate (PET-CF), providing foundational preliminary data for material selection and design [13] in low-energy environments.

The adoption of Additive Manufacturing (AM) [14], also known as 3D printing, represents a disruptive shift in the construction sector, offering unparalleled design freedom, material customization, and efficiency gains [15] over traditional methods. A key advantage of this technology is its ability to create complex geometries [16] and intricate internal structures, such as cellular or porous designs, which optimize functional properties like thermal insulation or mechanical strength. AM also offers immense potential for material innovation [17], allowing for tailored composites, such as carbon fiber-reinforced PET-CF [18]. Furthermore, AM enables the creation of functional gradients and controlled porosity—here qualitatively described as the engineered distribution of micro-voids during the foaming process to reduce the overall thermal conductivity—for enhanced insulation, as seen in PLA Aero [19,20] and multi-material components [21].

This technology inherently promotes more sustainable construction practices [22] through reduced material waste, on-demand production, and the use of recycled or bio-based feedstocks (e.g., PLA) [23]. For smart facades, 3D printing is particularly impactful [24], facilitating the fabrication of optimized thermal barriers, integrated functional elements like sensors or wiring directly within components, lightweight and high-performance components, and customizable aesthetics and forms with optimal energy performance [25].

However, realizing the full potential of these innovations requires understanding how printing parameters—such as layer height, raster orientation, and infill density—and material compositions translate into actual performance properties [26]. Critical issues remain unaddressed regarding the anisotropy of 3D-printed insulation and how the printing process alters the intrinsic polymer properties compared to bulk materials.

To ensure scientific clarity, this study distinguishes between effective thermal conductivity (λ_eff_), which characterizes the printed monolithic specimens (including process-induced micro-voids and raster interfaces), and apparent thermal conductivity (λ_app_), which describes the system-level performance of multi-layered assemblies (e.g., air-core structures).

The present study contributes to this understanding by systematically investigating the effective and apparent thermal conductivity of various 3D-printed polymeric materials, providing preliminary data for designing next-generation smart facade components.

The building envelope, encompassing walls, roof, and windows, serves as the primary boundary impacting energy efficiency and sustainable performance. Among the properties influencing this boundary, thermal conductivity is a fundamental characteristic directly impacting heat transfer [27]. It is essential to distinguish between effective thermal conductivity, which characterizes the 3D-printed monolithic specimens, and apparent thermal conductivity, which describes the performance of complex geometries or multi-layered systems. A lower conductivity value indicates superior insulating properties, directly correlating with reduced energy consumption for heating and cooling [28,29]. Beyond energy demands, the thermal performance of envelope materials profoundly affects indoor thermal comfort, mitigating risks of radiant temperature asymmetry, surface condensation, and mold growth [30,31]. Consequently, thermal conductivity guides architectural choices, from insulation thickness [32] to the mitigation of thermal bridges [33].

For the specific context of smart facades utilizing 3D-printed components, the effective management of apparent thermal conductivity is critical. Smart facades aim for dynamic thermal performance [8], necessitating reliable data for thermal conductance and thermal resistance (R-value) calculations. Since 3D printing enables novel geometries [34], the resulting thermal properties often differ from standard bulk material values due to engineered porosity and density variations. Detailed experimental data are therefore essential for advanced building energy simulation (BEM) and CFD analyses [35]. In summary, a systematic characterization of both effective and apparent thermal conductivity is essential to unlock the potential of 3D-printed components in creating energy-efficient and sustainable built environments.

The primary objective of this preliminary research is to contribute to bridging the gap between laboratory-scale additive manufacturing and the practical requirements of smart facade thermal design. Four polymers, a semi-crystalline polyester (polylactic acid—PLA Basic), a foamable semi-crystalline polyester (poly-L-lactic acid—PLA Aero), an amorphous polyester (polyethylene terephthalate glycol—PETG), and a carbon fiber-reinforced polyester (PET-CF), were investigated in this study. Specifically, we investigate how 3D printing parameters and material selection impact the thermal performance of potential facade components.

This study systematically evaluates the through-thickness thermal performance of four 3D-printed polymers and multi-layered air-core structures, specifically analyzing the impact of engineered micro-porosity in poly-L-lactic acid (PLA Aero) and carbon fiber-induced thermal bridging in polyethylene terephthalate (PET-CF) relative to conventional glazing benchmarks.

## 2. Materials and Methods

### 2.1. Investigated 3D-Printed Polymer Materials

In this study, four distinct polymers were investigated: a semi-crystalline polylactic acid (PLA Basic), a foamable poly-L-lactic acid (PLA Aero), an amorphous polyethylene terephthalate glycol (PETG), and a carbon fiber-reinforced polyethylene terephthalate (PET-CF). The reported thermophysical properties of these materials are listed in Table 1. The PLA-based and PET-CF filaments were sourced from Bambu Lab (Shenzhen, China), while the PETG (ReFill PETG) was provided by Formfutura (Nijmegen, The Netherlands). Specific batch numbers for each material are provided in the following descriptions.

- **PLA Basic (polylactic acid)**: The material used is a poly-L-lactic acid (PLLA) based polymer. While PLA is bio-based and biodegradable, its low heat deflection temperature (typically 50–60 °C) is a limitation for direct exposure on exterior facade surfaces. However, for building insulation, biodegradability may not be a required functional property; thus, it was selected for this study to evaluate its performance as a protected internal insulating core within multi-layered smart facade systems, where it remains shielded from direct environmental degradation and extreme surface temperatures. To ensure optimal processing and minimize moisture-induced defects during extrusion, the filament was dried at 45 °C for 6 h prior to printing using a dedicated filament drying station with forced air circulation.
- **PLA Aero (foaming grade)**: Confirmed as a PLLA-based material, this filament utilizes specialized active foaming additives that thermally decompose during the printing process. This creates a micro-porous, closed-cell internal structure, where the final porosity is controlled by the extrusion temperature and flow ratio. This engineered porosity is intended to reduce the effective thermal conductivity by substituting a portion of the solid polymer matrix with quiescent air pockets. To ensure material stability, the filament was dried at 45 °C for 6 h before printing. The resulting specimens were subsequently characterized to assess the semi-quantitative insulation benefits of this foamed architecture.
- **PETG (polyethylene terephthalate glycol)**: PETG was selected for its superior chemical resistance and higher Heat Deflection Temperature (HDT) compared to PLA, enhancing its stability under thermal stress in facade applications. To prevent depolymerization during the melt process and eliminate moisture-induced porosity, the filament was dried at 65 °C for 4 h prior to printing.
- **PET-CF (carbon fiber-reinforced PET)**: This technical-grade composite consists of a semi-crystalline PET matrix reinforced with 25 wt.% chopped carbon fibers. While the fibers enhance mechanical stability, they also potentially introduce thermal anisotropy and act as “thermal bridges,” increasing the effective conductivity. The filament was dried at 85 °C for 10 h before fabrication, following the manufacturer’s strict requirements to ensure optimal inter-layer bonding.

To establish a baseline for the materials investigated, the technical specifications provided by the manufacturers are summarized in Table 1. These thermophysical properties—including density, mechanical strength, and thermal resistance—serve as preliminary indicators for material selection in facade applications. It should be noted that while these values describe the raw filaments, the final thermal performance of the 3D-printed parts is further influenced by the FDM process parameters, which dictate the actual density and internal architecture of the specimens.

### 2.2. Fabrication of Samples for Thermal Analysis

The geometry of the 3D-printed specimens was engineered using Cadwork software V.29, ensuring control over the internal structures and external dimensions. The test plates (200 mm × 200 mm) were fabricated using a Bambu Lab A1 3D printer (Shenzhen, China), a high-precision Cartesian system equipped with a 0.4 mm stainless steel nozzle. To characterize the effective material properties, all specimens were configured with a 100% solid rectilinear infill pattern and a fine layer height of 0.12 mm. This strategy was selected to ensure continuous edge-to-edge raster deposition, thereby minimizing designed macroscopic structural voids. In the specific case of PLA Aero, while the 100% infill setting was maintained to ensure a continuous toolpath, the flow ratio was reduced to 0.6; this allowed the material to expand via its active foaming agents during extrusion, filling the volumetric space with a micro-porous matrix. Consequently, the observed porosity in PLA Aero is inherent to the material’s foaming morphology rather than programmed gaps in the print geometry. These process-induced micro-voids and foaming-induced pores are qualitatively discussed in the microstructural analysis (Section 2.5).

For the monolithic specimens, the 100% rectilinear infill was applied throughout the entire thickness. To ensure reproducibility, all samples were printed with 10 wall loops (perimeters) and a cross-ply raster orientation (±45°). The specimens were oriented flat on the build plate (XY orientation) so that the layer deposition was perpendicular to the heat flow direction during testing. Printing was conducted in a draft-free laboratory environment at a stable ambient temperature (~23 °C). Specific fabrication parameters, including actual print speeds and thermal settings, are detailed in Table 2.

### 2.3. Thermal Conductivity Measurement (GHP Method)

The experimental determination of thermal conductivity is based on Fourier’s Law, which describes steady-state heat conduction through a plane plate as $q=\frac{\lambda \cdot \Delta T}{\delta }$. Effective thermal conductivity (λ) is thus quantified as a thermophysical property [36] that depends on temperature and structural characteristics. To measure this property, we used the Feutron 4110 Guarded Hot Plate (GHP) apparatus, a precision instrument designed for the high-precision characterization of insulating and building materials.

To ensure strict adherence to international standards ASTM C177 [37], EN 12667 [38], and EN 12939 [39], the specimens (200 mm × 200 mm) were placed between a guarded hot plate and a cold plate enclosed by a heat insulation box. This configuration matches the maximum dimensions of the GHP plates, ensuring a strictly one-dimensional heat flux by eliminating lateral heat losses. Furthermore, as required by EN 12667 for thick products, the apparatus underwent a systematic calibration and verification procedure before each experimental session using a certified reference material—a glass sample with a known thermal conductivity of 1.15 W/m·K.

Steady-state conditions were confirmed once the temperature fluctuation across the plates remained below 0.05 K for a minimum period of 60 min. Sample thickness (δ) was measured using a digital micrometer (±0.01 mm) at multiple points both at the beginning and end of each test to account for any potential thermal deformation. The mean of these measurements provided the corrected thickness values for the final effective and apparent thermal conductivity calculations.

**Figure 2:**
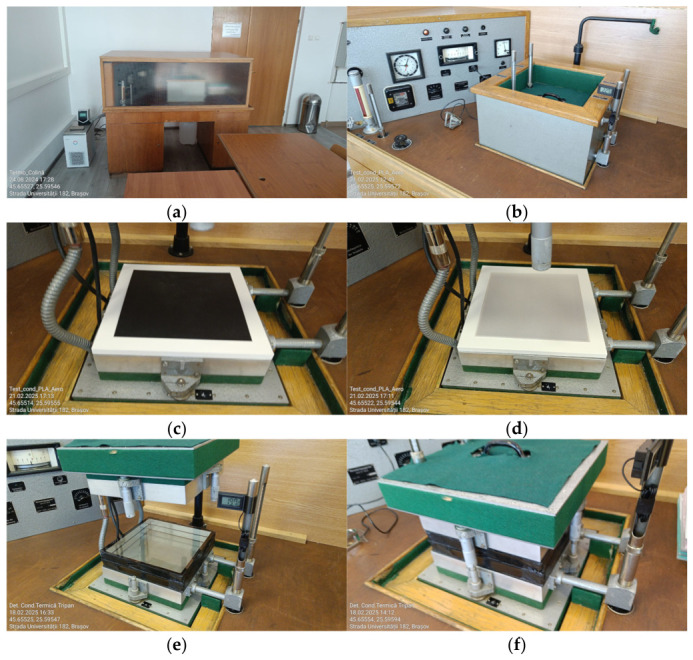
Experimental setup and specimen loading stages for thermal conductivity measurements: (**a**) general laboratory view of the Feutron 4110 GHP apparatus; (**b**) close-up of the control interface and sample chamber; (**c**) 3D-printed PET-CF specimen (200 mm × 200 mm); (**d**) placement of the monolithic PETG sample on the lower plate; (**e**) assembly of the multi-layered glass-air-glass system; (**f**) fully assembled GHP configuration ready for measurement.

The experimental setup, including the successive stages of specimen preparation and loading into the GHP chamber, is illustrated in Figure 2. To ensure the high-precision characterization required for these 3D-printed materials, each stage—from the placement of the monolithic plates to the assembly of multi-layered systems—was carefully monitored to maintain perfect contact between the specimen surfaces and the heat-controlled plates, thereby minimizing contact resistance.

As shown in Figure 2c,d, this configuration ensures a strictly one-dimensional heat flux by eliminating lateral heat losses, matching the maximum dimensions of the GHP heating and cooling plates. This alignment is critical for the accurate determination of both the effective thermal conductivity of monolithic plates and the apparent thermal conductivity of complex assemblies.

### 2.4. Multi-Layered Air-Core Structures and Glass Assemblies

**Figure 3:**
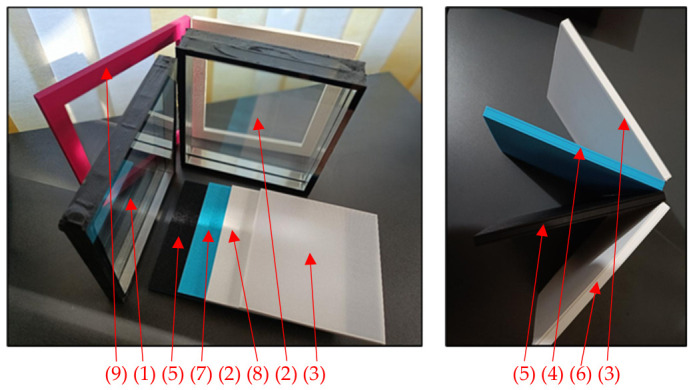
Visual representation of the eight investigated test specimens and assemblies: (1–2) multi-layered glass assemblies for apparent thermal conductivity reference; (3, 5, 7, 8) monolithic 3D-printed specimens for effective thermal conductivity characterization; (4, 6) 3D-printed air-core panels for system-level apparent performance evaluation; and (9) GHP guard ring. (Detailed key: (1) GLASS-AIR-GLASS; (2) GLASS-AIR-GLASS-AIR-GLASS; (3) monolithic PETG; (4) PLA-AIR-PLA; (5) monolithic PET-CF; (6) PLA Aero-AIR-PLA Aero; (7) monolithic PLA Basic; (8) monolithic PLA Aero).

The study extended the analysis to complex assemblies to evaluate potential facade applications. The complete set of the eight investigated test specimens, including monolithic plates and the multi-layered configurations, is shown in Figure 3. This set included 3D-printed air-core panels (PLA-AIR-PLA and PLA Aero-AIR-PLA Aero) and conventional glass assemblies (double and triple-glazed equivalents). The glass assemblies utilized standard 4 mm thick clear float glass panes (non-coated, emissivity ~0.84), separated by aluminum spacer bars to define the internal air cavities. The perimeter of these units was hermetically sealed using professional-grade hot-melt butyl rubber sealant, ensuring a quiescent air gap representative of industrial insulating glass unit (IGU) technology.

The 3D-printed air-core panels were designed with 2 mm thick external faces (printed with 100% solid infill) to enclose the internal air gaps. These assemblies were designed to evaluate the apparent thermal conductivity (λ_app_) of the system. The geometric accuracy of all assemblies was verified through thickness measurements. Table 3 summarizes corrected assembly thicknesses and the resulting internal air gaps, providing the necessary data to distinguish between effective material properties and apparent system-level thermal performance The thermal performance of the investigated assemblies is quantified by their thermal conductance (U=q/ΔT) and the corresponding thermal resistance (R). It is important to emphasize that these values characterize the behavior under specific steady-state laboratory conditions and should not be confused with standardized building-envelope U-values or total R-values. Unlike official building metrics, these results focus on the intrinsic thermophysical performance of the 3D-printed geometries and air-core structures, as surface resistances were not included in this characterization.

### 2.5. Microstructural Characterization Method

**Figure 4:**
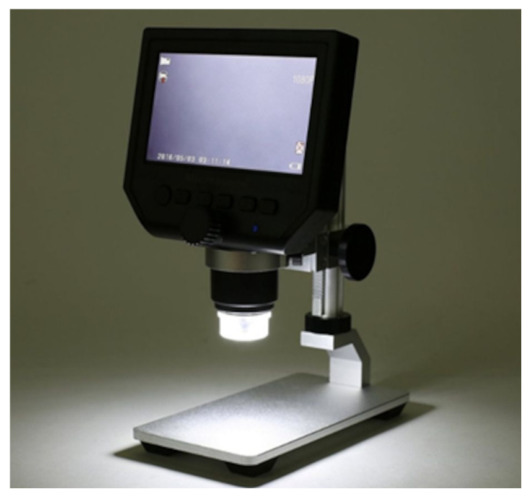
Digital FullHD 1-600X microscope setup used for the microstructural documentation of the 3D-printed specimens.

To qualitatively investigate the internal morphology and porosity resulting from the printing process, a Digital FullHD 1-600X microscope (View Solutions Inc., Nanjing, China) was employed (Figure 4). The objective was to visually verify the outcome of the 100% infill setting, ensuring that the rasters were deposited with consistent edge-to-edge contact. This instrument, equipped with a 3.6 MP camera, provided high-resolution imaging of the internal layers. While the microstructural examination was primarily focused on the morphological observation of the foamed architecture in PLA Aero, all specimens were inspected to confirm infill consistency and the absence of macroscopic internal defects. To ensure the integrity of the micro-pores and avoid mechanical deformation caused by traditional cutting, a ‘mid-print sampling’ technique was utilized, pausing the printing process at 50% completion for direct observation of the undisturbed internal structure. This assessment provides visual evidence of the micro-porous distribution, supporting the effective thermal conductivity results without deriving statistical porosity data.

Image processing and spatial calibration were performed using Fiji (an enhanced ImageJ distribution, version 2.1.3; https://fiji.sc/, accessed on 2 April 2026). The software was calibrated by converting pixel distances into real units (µm) based on a known reference dimension, allowing for the precise application of the scale bars shown in the Section 3.

### 2.6. Uncertainty Analysis

A systematic uncertainty analysis was performed following the ISO/IEC Guide 98-3 (GUM) [40] to ensure the reliability of the experimental data. The combined standard uncertainty $u_{c\lambda }$ for the thermal conductivity was evaluated by considering both Type A (statistical) and Type B (systematic) contributions.

The combined uncertainty was calculated using the law of propagation of uncertainty applied to Fourier’s Law equation $q=\lambda \cdot \frac{\Delta T}{\delta }$:

$$\frac{u_{c\left(\lambda \right)}}{\lambda }=\sqrt{\left({\left(\frac{u\left(q\right)}{q}\right)}^{2}+{\left(\frac{u\left(\delta \right)}{\delta }\right)}^{2}+{\left(\frac{u\left(\Delta T\right)}{\Delta T}\right)}^{2}\right)},$$

The primary sources of Type B uncertainty included the calibration of the Feutron 4110 sensors (Langenwetzendorf, Germany) $u\left(T\right)=\pm 0.05\;K$, the digital micrometer precision $u\left(\delta \right)=\pm 0.01\;\mathrm{mm}$, and the electrical power measurement for the heat flux $\frac{u\left(q\right)}{q}=\pm 0.5$**%**. All uncertainty calculations and data processing were performed using Microsoft Excel (Version 2108, Build 14334.20756; Microsoft Corp., Redmond, WA, USA; https://www.microsoft.com/excel, accessed on 1 February 2026). Type A uncertainty $u_{A\left(\lambda \right)}$ was determined through three independent repeated measurements for each sample type to account for material inhomogeneity and process repeatability. The combined standard uncertainty is thus calculated as:

$$u_{c}=\sqrt{u_{A}^{2}+u_{B}^{2}}$$

The expanded uncertainty (U) was calculated using a coverage factor of k = 2, providing a confidence level of approximately 95% $\left(U=k\cdot u_{c}\right)$. Based on this analysis, the total uncertainty for the reported λ_eff_ and λ_app_ values was estimated to be within ±3.2% to ±4.8%. Consequently, all numerical results in Section 3 are reported with the appropriate number of significant figures, harmonized with the calculated expanded uncertainty intervals.

## 3. Results

The experimental assessment of the thermal properties for both monolithic specimens and multi-layered assemblies was conducted under steady-state conditions. The primary measured quantities—including corrected thickness (δ), heat flux (q), and temperature drop (ΔT)—as well as the calculated values for thermal conductivity (λ_eff_, λ_app_) and thermal resistance (R), which quantifies the material’s opposition to heat flow, are summarized in Table 4. To ensure scientific consistency and reflect the uncertainty analysis (Section 2.6), the numerical precision of each parameter has been harmonized with its respective uncertainty interval. While thermal conductivity values are reported to three decimal places, quantities such as heat flux and temperature drop have been rounded to reflect the instrumental and statistical limits of the experimental setup. All results are reported with their respective standard deviations and expanded uncertainty intervals (k = 2).

The reported expanded uncertainty intervals (k = 2) encompass the statistical standard deviation from the three repetitions (Type A) and the systematic (Type B) errors, following the uncertainty budget established in Section 2.6.

**Figure 5:**
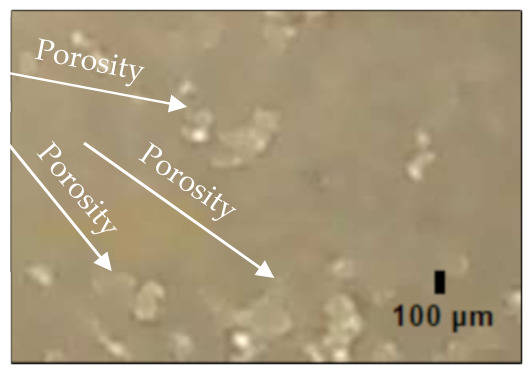
Cross-sectional optical microscopy view of a 3D-printed PLA Aero panel, providing a qualitative illustration of the micro-porous internal structure and trapped air pockets resulting from the expansion of foaming agents during the FDM process.

The effective thermal conductivity of 3D-printed PLA Basic (0.267 W/(m·K)) provided a reference baseline. In contrast, PLA Aero exhibited a significantly reduced conductivity of 0.114 W/(m·K), representing a 57.3% reduction. This insulation gain is directly linked to the engineered micro-porosity, which was qualitatively observed in Figure 5. This porous structure is a direct result of the chemical foaming agents within the PLA Aero filament, which expand during the extrusion process as the polymer leaves the nozzle. The examination reveals a distinctive network of irregular micro-pores (typically visually estimated in the range of 10 to 50 µm) distributed within the matrix, acting as thermal barriers. This morphological assessment provides a semi-quantitative understanding of the insulation benefits, supporting the measured reduction in the overall effective thermal conductivity.

PETG showed an effective conductivity of 0.290 W/(m·K), while the carbon-fiber-reinforced PET-CF demonstrated the highest values at 0.533 W/(m·K), indicating the thermal bridging effect of the reinforcement. Regarding multi-layered systems, the PLA Aero-AIR-PLA Aero assembly achieved the lowest apparent thermal conductivity at 0.051 W/(m·K), outperforming both the 3D-printed PLA-AIR-PLA (0.080 W/(m·K)) and the conventional double-glazed glass unit (0.058 W/(m·K)).

The conventional glass benchmarks (summarized in Table 4) resulted in measured thermal conductance $\left(\frac{q}{\Delta T}\;\right)$ values of approximately 2.34 W/(m^2^·K) for the double-glazed unit and 1.71 W/(m^2^·K) for the triple-glazed configuration. It is important to clarify that these values represent the conductance under the specific GHP test configuration and should not be directly equated with standardized building-envelope U-values, as they do not incorporate standard surface resistances. These preliminary quantitative foundations, including a brief discussion of the R-values in Table 4, serve as the basis for the comparative performance analysis discussed in Section 4.

## 4. Discussion

It is crucial to distinguish between the effective thermal conductivity (λ_eff_) of 3D-printed monolithic materials (PLA Basic, PLA Aero, PETG, PET-CF) and the apparent thermal conductivity (λ_app_) of the multi-layered systems. While the Guarded Hot Plate (GHP) method treats these complex assemblies as homogeneous entities for practical engineering applications, the resulting values represent a system-level performance that integrates contributions from polymer layers, interfaces, and quiescent air gaps.

Furthermore, it is important to clarify that the measured values for monolithic samples represent the effective conductivity of the printed specimens—including process-induced features like raster structures, interfaces, and micro-porosity—rather than the properties of the bulk polymer pellets. This distinction is essential, as the FDM process inherently alters the material’s thermal behavior compared to its raw form.

**Figure 6:**
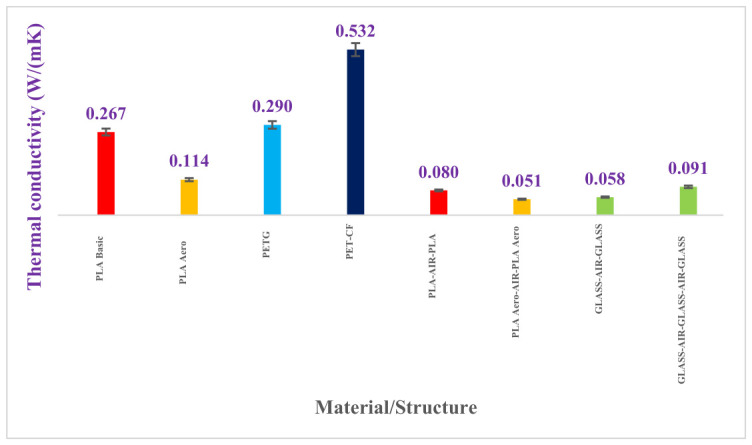
Comparative thermal conductivity of investigated 3D-printed materials and structures. Error bars represent the expanded uncertainty (U) with a coverage factor k = 2.

To commence the comparative analysis, Figure 6 presents an evaluation of the thermal conductivity for all eight investigated materials and structural configurations. In accordance with the uncertainty analysis, Figure 6 incorporates error bars corresponding to the expanded uncertainty (k = 2) for each measurement, ensuring a transparent comparison of the performance across different specimens.

The experimental data reveals a wide distribution of thermal performance, ranging from an apparent thermal conductivity of 0.051 W/(m·K) for optimized air-core structures to an effective thermal conductivity of 0.533 W/(m·K) for reinforced composites.

The measured effective conductivity for 3D-printed PLA Basic (0.267 W/(m·K)) is notably higher than the values of 0.13–0.20 W/(m·K) typically reported for unprocessed pellets [41,42]. This discrepancy is attributed to the FDM process, where the melting and subsequent fusion of the polymer layers create a more continuous and consolidated matrix compared to compressed pellets, which inherently contain insulating micro-voids. Furthermore, the 100% infill strategy used in this study, aiming for maximum edge-to-edge raster contact, combined with the thermal history during printing, may influence crystallinity levels and potentially enhance heat transfer pathways within the polymer matrix [43]. These observations confirm that the effective thermal properties are a result of both the base material and the specific additive manufacturing process.

In contrast, PLA Aero demonstrated a 57.3% reduction in effective conductivity (0.114 W/(m·K)) compared to its basic counterpart. This result aligns with findings for foamed PLA variants (0.05–0.15 W/(m·K)) found in the recent literature [44]. The engineered micro-porosity, qualitatively observed in the morphological analysis (Figure 5), acts as a series of thermal barriers, effectively replacing the solid polymer with low-conductivity air pockets. This porous structure is a direct result of the chemical foaming agents within the PLA Aero filament, which expand as the polymer leaves the nozzle during the extrusion process. This confirms that for potential facade applications, the qualitative control of internal morphology via 3D printing parameters (such as flow ratio) is as critical as the material selection itself.

### 4.1. Influence of Reinforcements and Composite Morphology: PETG vs. PET-CF

The comparison between monolithic PETG and carbon-fiber-reinforced PET-CF reveals a substantial 83.5% increase in effective thermal conductivity, rising from 0.290 W/(m·K) to 0.532 W/(m·K). However, it is important to note that a direct comparison between these two materials is limited by their differing polymer morphologies: PETG is an amorphous copolymer, whereas the matrix in PET-CF is a semi-crystalline polyester [45,46].

Our measured value for PETG (0.290 W/(m·K)) aligns closely with the upper range of 0.2–0.3 W/(m·K) reported in the literature for neat filaments, although it is higher than the 0.215 W/(m·K) observed by Valvez et al. [45] in specific FDM optimization studies. This difference is attributed to the high layer bonding achieved through our specific printing parameters (0.12 mm layer height and 100% rectilinear infill), which minimize contact resistance between layers and reduce process-induced porosity.

The sharp increase observed in PET-CF (0.532 W/(m·K)) is primarily attributed to the “thermal bridging” effect of the chopped carbon fibers. This value falls within the typical range of 0.5–2.0 W/(m·K) reported for carbon-reinforced polymer composites [46], reflecting the specific fiber loading (25 wt.%) and the semi-crystalline nature of the PET matrix, which supports higher thermal transport compared to amorphous structures.

Crucially, the reported value of 0.532 W/(m·K) represents the performance measured primarily in the through-thickness direction. While fiber alignment during extrusion typically induces thermal anisotropy [43], no in-plane measurements were performed in this study; thus, conclusions regarding distinct anisotropic ratios remain hypothetical and require further investigation.

### 4.2. Comparative Performance of Multi-Layered Air-Core Assemblies

**Figure 7:**
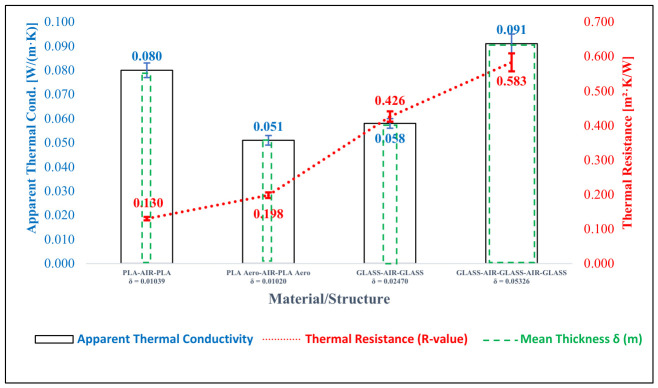
Comparison of apparent thermal conductivity (λ_app_) and thermal resistance (R-value) for multi-layered air-core structures. Note: These quantities are plotted on separate axes to distinguish their different physical units and meanings. Error bars represent the expanded uncertainty (k = 2).

The comparative analysis of multi-layered structures, as illustrated in Figure 6 and Figure 7, highlights the critical role of air layers in enhancing thermal insulation. The experimental results reveal that the PLA Aero-AIR-PLA Aero system achieved the lowest apparent thermal conductivity (λ_app_ = 0.051 W/(m·K)), outperforming both the standard PLA-AIR-PLA panel (0.080 W/(m·K)) and the conventional double-glazed glass assembly (0.058 W/(m·K)).

This performance is attributed to a “synergy effect”: the micro-porosity within the 2 mm PLA Aero faces inhibits heat conduction, while the 6.15 mm quiescent air gap acts as a thermal barrier. In contrast, the GLASS-AIR-GLASS assembly, despite its 16.6 mm air gap, exhibited a slightly higher λ_app_. These results demonstrate that engineered polymer-air systems can compete with conventional glazing in terms of apparent thermal performance.

A strictly hypothetical explanation is provided for the result observed for the triple-glazed unit (GLASS-AIR-GLASS-AIR-GLASS), which exhibited a higher λ_app_ (0.091 W/(m·K)) than the double-glazed variant (0.058 W/(m·K)). While the total thermal resistance (R-value) is higher for the thicker triple-glazed system (Table 4), the apparent conductivity is sensitive to the cavity geometry. It is hypothesized that larger or multiple air interstices may facilitate increased convective heat transfer or radiative effects, although no direct evidence of air movement or emissivity contributions was measured to confirm this mechanistic conclusion.

To further evaluate the insulation efficiency for building envelope applications, Figure 7 illustrates the relationship between apparent thermal conductivity and total thermal resistance (R-value) for the multi-layered structures. In accordance with the Reviewer’s recommendation, these two quantities are presented on separate axes (or sub-plots) to avoid suggesting direct physical equivalence between parameters with different units and physical meanings. This dual-parameter visualization highlights the synergy between 3D-printed micro-porosity and macroscopic air gaps, while maintaining a clear distinction between the system’s rate of heat transfer (λ_app_) and its overall insulating capacity (R-value).

The measured conductance (2.34 W/(m^2^K) for double glazing and 1.71 W/(m^2^K) for triple glazing) aligns with established calculation methodologies for glazing systems [47,48]. However, these should not be confused with full building-envelope U-values, as surface resistances were not included. To put these results into a broader context, although the apparent conductivity of the optimized PLA Aero system (0.051 W/(m·K)) is higher than that of specialized insulation like EPS (~0.035 W/(m·K)), it represents a significant advancement for structural polymer components that combine load-bearing potential with enhanced thermal resistance.

### 4.3. Practical Implications for Smart Facades and Study Limitations

The experimental data provided in this study serve as a preliminary thermophysical database for the potential future design of 3D-printed facade components. However, the application of these laboratory-scale results to real-world building envelopes must be approached with caution. Specifically, the tested samples represent passive thermal specimens only; this study does not experimentally demonstrate active smart facade functionality, such as adaptive behavior, integrated sensing, or control unit implementation. The measurements were conducted under steady-state conditions at a single mean temperature (~25–27 °C). In contrast, real facade systems are subject to highly dynamic environmental stressors, including significant temperature gradients, solar radiation, wind pressure, and humidity fluctuations.

Furthermore, the long-term durability of the investigated polymers remains a critical limitation. PLA and PLA Aero, while exhibiting excellent insulation properties, possess low heat deflection temperatures and are susceptible to UV degradation and moisture absorption if exposed directly to the outdoor environment. Consequently, these materials are best suited for internal insulating core layers, protected by durable external skins such as glass or high-performance, weather-resistant polymers. This aligns with our findings regarding the apparent thermal conductivity (λ_app_) of multi-layered systems, where the printed core is shielded by external panes.

Future research should focus on “scaling up” these preliminary findings through full-scale prototyping and Computational Fluid Dynamics (CFD) simulations that account for transient thermal behavior and long-term thermal aging. These future studies should also explore actual smart facade functionalities, including adaptive thermal regulation and active control systems. While our study supports the applicability of 3D-printed air-core structures for enhanced insulation, practical implementation will require additional testing regarding fire behavior, structural creep, and freeze–thaw resistance.

## 5. Conclusions

This research provided a systematic experimental investigation into the effective and apparent thermal conductivity of diverse 3D-printed polymers and multi-layered air-core structures. By adhering to international standards and performing a preliminary uncertainty analysis that accounts for both Type A and Type B contributions, this study established a reliable thermophysical database for the development of energy-efficient facade components.

A key finding is PLA Aero’s remarkable 57.3% reduction in effective thermal conductivity (0.114 W/(m·K)) compared to PLA Basic (0.267 W/(m·K)), highlighting the insulation potential of engineered micro-porosity. Conversely, PET-CF exhibited an 83.5% increase in conductivity compared to PETG, underscoring the trade-off where carbon fibers enhance mechanical properties but act as “thermal bridges,” compromising insulating capacity. The consistency of these experimental data with the literature, considering factors like polymer crystallinity and FDM-induced morphology, supports the applicability of the GHP methodology for characterizing additively manufactured materials.

Multi-layered structures with air gaps proved highly effective, with the PLA Aero-AIR-PLA Aero system achieving the lowest apparent thermal conductivity (0.051 W/(m·K)) and a corresponding R-value of 0.198 m^2^K/W. The analysis emphasized the R-value as a more reliable metric for building physics, noting that air’s insulating effectiveness is hypothesized to depend heavily on cavity geometry to prevent convective or radiative heat transfer, although further mechanistic studies are needed. Furthermore, the optimization of printing parameters (density, infill pattern, and layer height) was shown to be crucial for tailoring the internal architecture and subsequent thermal properties.

In conclusion, this research provides preliminary thermophysical data essential for material selection and the optimization of next-generation facade designs. These results provide a foundation to enhance the accuracy of Building Energy Simulation (BEM) and CFD analyses, demonstrating the potential role of 3D printing in the construction sector.

Despite these advancements, the current study is limited to laboratory-scale, steady-state conditions using passive materials. Future research must address critical factors such as scaling to full-sized components, environmental durability (UV stability, moisture absorption), and long-term thermal aging. Further investigation into potential thermal anisotropy and dynamic temperature dependency, along with the potential integration of active sensors and control systems, will be necessary to fully exploit 3D-printed materials for real-world smart facade applications.

## Figures and Tables

**Table 1:** Summarizes the available thermophysical properties for the filaments as provided by the manufacturers.

Filament Type	Manufacturer	Density (kg/m^3^)	Tensile Strength (MPa)	Impact Strength (kJ/m^2^)	Other Properties
Bambu PLA Basic	Bambu Lab	~1240	35 (X-Y), 31 (Z)	26.6 (X-Y), 13.8 (Z)	High toughness, Biodegradable
Bambu PLA Aero	Bambu Lab	~600–900 *	24 ± 2 (X-Y), 18 ± 3 (Z)	28.8 (X-Y), 8.2 (Z)	Lightweight, Foaming grade
ReFill PETG	Formfutura	1270	50 (Yield)	7.2 (Notched)	Amorphous, High HDT
Bambu PET-CF	Bambu Lab	~1320	74 (X-Y), 35 (Z)	36 (X-Y), 4.5 (Z)	Carbon-reinforced, Heat resistant

* Note: Density for PLA Aero varies based on the foaming expansion factor, which is controlled by the extrusion temperature and flow ratio during the FDM process. The apparent density of the actual printed specimens is reported in Section 2.2.

**Table 2:** Comprehensive FDM printing parameters for the investigated specimens.

Parameter	PET-CF	PETG	PLA Aero	PLA Basic
Nozzle Diameter	0.4 mm	0.4 mm	0.4 mm	0.4 mm
Nozzle Temp. (°C)	270	250	220	220
Bed Temp. (°C)	80	70	65	65
Flow Ratio	1.0	0.95	0.6	0.98
Infill Pattern/Density	100% Rect.	100% Rect.	100% Rect.	100% Rect.
Layer Height (mm)	0.12	0.12	0.12	0.12
Line Width (mm)	0.42	0.42	0.42	0.42
Wall loops (Perimeters)	10	10	10	10
Outer Wall Speed	60 mm/s	60 mm/s	60 mm/s	60 mm/s
Inner Wall Speed	150 mm/s	150 mm/s	150 mm/s	150 mm/s
Travel Speed	700 mm/s	700 mm/s	700 mm/s	700 mm/s

**Table 3:** Geometric specifications and thickness measurements for multi-layered assemblies.

Specimen Type (Assembly)	Component Thickness (mm)	Total Corrected Thickness δ (mm)	Calculated Internal Air Gap (mm)
PLA-AIR-PLA	2.00 (3D-printed)	10.35	~6.35
PLA Aero-AIR-PLA Aero	2.00 (3D-printed)	10.15	~6.15
GLASS-AIR-GLASS	4.00 (Glass)	24.66	~16.66
GLASS-AIR-GLASS-AIR-GLASS	4.00 (Glass)	53.21	~16.60 + 16.60 *

* Note: For the triple-glazed unit, the two air gaps are separated by a median glass pane. The total corrected thickness represents the mean value used for apparent thermal conductivity calculations.

**Table 4:** Centralized experimental results for monolithic specimens and complex assemblies.

Specimen/ Assembly	Measured Density (kg/m^3^)	Mean Thickness δ (m)	Heat Flux q (W/m^2^)	ΔT (K)	Thermal Cond. (W/(m·K))	R-Value (m^2^·K/W)
Monolithic Samples					λ_eff_	
PLA Basic	1240	0.01019	260.4	10.21	0.267 ± 0.011	0.039
PLA Aero	740 *	0.01094	70.4	6.83	0.114 ± 0.005	0.097
PETG	1270	0.01014	279.8	9.77	0.290 ± 0.012	0.034
PET-CF	1320	0.01007	398.7	7.50	0.533 ± 0.021	0.018
Complex Assemblies					λ_app_	
PLA-AIR-PLA	N/A	0.01039	49.9	6.50	0.080 ± 0.003	0.130
PLA Aero-AIR-PLA Aero	N/A	0.01020	55.8	11.14	0.051 ± 0.002	0.198
GLASS-AIR-GLASS	N/A	0.02470	14.5	6.21	0.058 ± 0.002	0.426
GLASS-AIR-GLASS- AIR-GLASS	N/A	0.05326	18.7	10.93	0.091 ± 0.004	0.583

* Note: For PLA Aero, the density is an apparent value resulting from the 0.6 flow ratio expansion.

## Data Availability

The original contributions presented in this study are included in the article. Further inquiries can be directed to the corresponding author.

## Abbreviations

The following abbreviations are used in this manuscript:

AM	Additive Manufacturing (3D printing)
ASTM	American Society for Testing and Materials
BEM	Building Energy Modeling
CFD	Computational Fluid Dynamics
EN	European Standard
FDM	Fused Deposition Modeling
GHP	Guarded Hot Plate (guarded-hot-plate method)
GLASS	Glass (used in multi-layer configurations, e.g., GLASS-AIR-GLASS)
GUM	Guide to the Expression of Uncertainty in Measurement
HDT	Heat Deflection Temperature
HVAC	Heating, Ventilation, and Air Conditioning
IGU	Insulating Glass Unit
ISO	International Organization for Standardization
PETG	Polyethylene Terephthalate Glycol
PET-CF	PET reinforced with Carbon Fiber
PLA	Polylactic Acid (PLA Basic)
PLA Aero	Polylactic Acid (foaming/foamed grade)
PLLA	Poly-L-lactic acid
q	heat flux [W/m^2^]
R-value	thermal resistance [m^2^·K/W]
u_c_	combined standard uncertainty
U-value	thermal transmittance (conductance) [W/(m^2^·K)]
U	expanded uncertainty ($k = 2$)
δ	sample thickness [m]
ΔT	temperature difference [K]
λ_eff_	effective thermal conductivity (for monolithic specimens) [W/(m·K)]
λ_app_	apparent thermal conductivity (for multi-layered assemblies) [W/(m·K)]
ZEB	Zero-Emission Building

## References

[1] Firoozi A.A., Firoozi A.A., Oyejobi D.O., Avudaiappan S., Flores E.S. (2024). Emerging trends in sustainable building materials: Technological innovations, enhanced performance, and future directions. Results Eng..

[2] Tan Y., Shen L., Yao H. (2011). Sustainable construction practice and contractors’ competitiveness: A preliminary study. Habitat Int..

[3] Pérez-Lombard L., Ortiz J., Pout C. (2008). A review on buildings energy consumption information. Energy Build..

[4] Yaman M. (2021). Different facade types and building integration in energy efficient building design strategies. Int. J. Built Environ. Sustain..

[5] Perino M., Serra V. (2015). Switching from static to adaptable and dynamic building envelopes: A paradigm shift for the energy efficiency in buildings. J. Facade Des. Eng..

[6] Sartori I., Hestnes A.G. (2007). Energy use in the life cycle of conventional and low-energy buildings: A review article. Energy Build..

[7] Baraboi D.-R., Scutaru L.M., Dragomir G., Brezeanu A.I., Calotă R., Pavel M., Năstase G. (2025). Smart 3D-printed facades: A review of innovations, materials, and sustainable performance. Front. Sustain. Cities.

[8] Wang J., Li S., Ye P. (2025). Dynamic Skin: A Systematic Review of Energy-Saving Design for Building Facades. Buildings.

[9] Murtagh N., Scott L., Fan J. (2020). Sustainable and resilient construction: Current status and future challenges. J. Clean. Prod..

[10] Chi D.A., Moreno D., Navarro J. (2017). Design optimisation of perforated solar façades in order to balance daylighting with thermal performance. Build. Environ..

[11] Grimvall G. (1999). Thermophysical Properties of Materials.

[12] Guo Y., Ruan K., Gu J. (2021). Controllable thermal conductivity in composites by constructing thermal conduction networks. Mater. Today Phys..

[13] Firoozi A.A., Firoozi A.A. (2023). Smart facades in architecture: Driving energy efficiency and adaptive urban design. SSRN.

[14] Wong K.V., Hernandez A. (2012). A review of additive manufacturing. Int. Sch. Res. Not..

[15] Oropallo W., Piegl L.A. (2016). Ten challenges in 3D printing. Eng. Comput..

[16] Teizer J., Blickle A., King T., Leitzbach O., Guenther D. (2016). Large scale 3D printing of complex geometric shapes in construction. Isarc, Proceedings of the International Symposium on Automation and Robotics in Construction, Montreal, QC, Canada, 28–31 July 2025.

[17] Borthakur P.P. (2025). The Role and Future Directions of 3D Printing in Custom Prosthetic Design. Eng. Proc..

[18] Lee C.H., Padzil F.N.B.M., Lee S.H., Ainun Z.M.A., Abdullah L.C. (2021). Potential for natural fiber reinforcement in PLA polymer filaments for fused deposition modeling (FDM) additive manufacturing: A review. Polymers.

[19] Markin V., Nerella V.N., Schröfl C., Guseynova G., Mechtcherine V. (2019). Material design and performance evaluation of foam concrete for digital fabrication. Materials.

[20] Rathore P.K.S., Aleem A., Sikarwar B.S., Sharma R.K., Kumar R., Gupta N.K. (2025). Experimental and simulation study of lightweight roof with thermal energy storage for energy-efficient building envelope. Therm. Sci. Eng. Prog..

[21] Gao Z., Yin J., Liu P., Li Q., Zhang R., Yang H., Zhou H. (2023). Simultaneous multi-material embedded printing for 3D heterogeneous structures. Int. J. Extrem. Manuf..

[22] Singh R., Gehlot A., Akram S.V., Gupta L.R., Jena M.K., Prakash C., Singh S., Kumar R. (2021). Cloud manufacturing, internet of things-assisted manufacturing and 3D printing technology: Reliable tools for sustainable construction. Sustainability.

[23] Morales M.A., Maranon A., Hernandez C., Michaud V., Porras A. (2023). Colombian sustainability perspective on fused deposition modeling technology: Opportunity to develop recycled and biobased 3D printing filaments. Polymers.

[24] Abid M.T., Khan S.A., Koç M. (2025). 3D printing in facilities management: A systematic review toward smart and sustainable building operations. Buildings.

[25] Mahdavinejad M., Bazazzadeh H., Mehrvarz F., Berardi U., Nasr T., Pourbagher S., Hoseinzadeh S. (2024). The impact of facade geometry on visual comfort and energy consumption in an office building in different climates. Energy Rep..

[26] Baraboi D.-R., Năstase G., Sima R., Șerban A. (2026). Evolution of Colorimetry in 3D-Printed Samples Exposed to External Weather Conditions, Used in Smart Façades. Buildings.

[27] D’Alessandro G., Potenza M., Corasaniti S., Sfarra S., Coppa P., Bovesecchi G., de Monte F. (2022). Modeling and measuring thermodynamic and transport thermophysical properties: A review. Energies.

[28] Haj Hussein M., Monna S., Abdallah R., Juaidi A., Albatayneh A. (2022). Improving the thermal performance of building envelopes: An approach to enhancing the building energy efficiency code. Sustainability.

[29] Lei J., Yang J., Yang E.-H. (2016). Energy performance of building envelopes integrated with phase change materials for cooling load reduction in tropical Singapore. Appl. Energy.

[30] Wang X., Sun X., Yu C.W. (2018). Building envelope with variable thermal performance: Opportunities and challenges. Indoor Built Environ..

[31] Lopez-Carreon I., Jahan E., Yari M.H., Esmizadeh E., Riahinezhad M., Lacasse M., Xiao Z., Dragomirescu E. (2025). Moisture Ingress in Building Envelope Materials:(II) Transport Mechanisms and Practical Mitigation Approaches. Buildings.

[32] Zheng K., Cho Y.K., Wang C., Li H. (2016). Noninvasive Residential Building Envelope R-Value Measurement Method Based on Interfacial Thermal Resistance. J. Archit. Eng..

[33] Anwajler B., Szołomicki J., Noszczyk P., Baryś M. (2024). The potential of 3D printing in thermal insulating composite materials—Experimental determination of the impact of the geometry on thermal resistance. Materials.

[34] Fakhr Ghasemi A., Pinto Duarte J. (2025). A Systematic Review of Innovative Advances in Multi-Material Additive Manufacturing: Implications for Architecture and Construction. Materials.

[35] Lü X., Lu T., Yang T., Salonen H., Dai Z., Droege P., Chen H. (2021). Improving the energy efficiency of buildings based on fluid dynamics models: A critical review. Energies.

[36] Tritt T.M. (2005). Thermal Conductivity: Theory, Properties, and Applications.

[37] (2019). Standard Test Method for Steady-State Heat Flux Measurements and Thermal Transmission Properties by Means of the Guarded-Hot-Plate Apparatus.

[38] (2001). Thermal Performance of Building Materials and Products. Determination of Thermal Resistance by Means of Guarded Hot Plate and Heat Flow Meter Methods. Products of High and Medium Thermal Resistance.

[39] (2000). Thermal Performance of Building Materials and Products—Determination of Thermal Resistance by Means of Guarded Hot Plate and Heat Flow Meter Methods—Thick Products of High and Medium Thermal Resistance.

[40] (2008). Uncertainty of Measurement—Part 3: Guide to the Expression of Uncertainty in Measurement (GUM:1995).

[41] MacFarland N., Carbajal G., Romero-Ramirez E., Stanfill C. (2024). Experimental Evaluation of the Thermal Conductivity of 3D-Printed Polylactic Acid Composite Materials. Proceedings of the Volume 9: Heat Transfer and Thermal Engineering, Portland, OR, USA, 17–21 November 2024.

[42] Ultimaker Ultimaker PLA Filament Material Data Sheet. Ultimaker, Technical Data Sheet. https://www.matweb.com/search/DataSheet.aspx?MatGUID=16027c449339463b90558b73f75e7a89.

[43] Blanco I., Cicala G., Recca G., Tosto C. (2022). Specific heat capacity and thermal conductivity measurements of PLA-based 3D-printed parts with milled carbon fiber reinforcement. Entropy.

[44] Sovetova M., Calautit J.K. (2024). Influence of printing parameters on the thermal properties of 3D-printed construction structures. Energy.

[45] Valvez S., Silva A.P., Reis P.N. (2022). Optimization of printing parameters to maximize the mechanical properties of 3D-printed PETG-based parts. Polymers.

[46] Alshammari B.A., Alsuhybani M.S., Almushaikeh A.M., Alotaibi B.M., Alenad A.M., Alqahtani N.B., Alharbi A.G. (2021). Comprehensive Review of the Properties and Modifications of Carbon Fiber-Reinforced Thermoplastic Composites. Polymers.

[47] (2011). Glass in Building—Determination of Thermal Transmittance (U Value)—Calculation Method.

[48] (1994). Glass in Building—Calculation of Steady-State U Values (Thermal Transmittance) of Multiple Glazing.

